# Integrase-derived peptides together with CD24-targeted lentiviral particles inhibit the growth of CD24 expressing cancer cells

**DOI:** 10.1038/s41388-021-01779-5

**Published:** 2021-05-06

**Authors:** Shiran Shapira, Eynat Finkelshtein, Dina Kazanov, Esmira Naftali, Irena Stepansky, Abraham Loyter, Daniel Elbirt, Mori Hay-Levy, Eli Brazowski, Faina Bedny, Roy Dekel, Dov Hershkovitz, Arye Blachar, Ido Wolf, Nadir Arber

**Affiliations:** 1 Health Promotion Center and Integrated Cancer Prevention Center, Sourasky Medical Center, Tel-Aviv, Israel; 2 Sackler Faculty of Medicine, Tel Aviv University, Tel-Aviv, Israel; 3 Zion Medical B.V, De Bilt, Netherlands; 4 Oncology Division, Tel Aviv Medical Center, Tel-Aviv, Israel; 5 Department of Biological Chemistry, The Alexander Institute of Life Sciences, Hebrew University, Jerusalem, Israel; 6 Clinical Immunology, Allergy and AIDS Center Kaplan Medical Center, Affiliated with Hadassah-Hebrew University Medical School Jerusalem, Rehovot, Israel; 7 Pathology Institute, Tel Aviv Sourasky Medical Center, Tel-Aviv, Israel; 8 Department of Radiology, Tel Aviv Sourasky Medical Center, Tel-Aviv, Israel

**Keywords:** Targeted therapies, Drug safety

## Abstract

The integration of viral DNA into the host genome is mediated by viral integrase, resulting in the accumulation of double-strand breaks. Integrase-derived peptides (INS and INR) increase the number of integration events, leading to escalated genomic instability that induces apoptosis. CD24 is a surface protein expressed mostly in cancer cells and is very rarely found in normal cells. Here, we propose a novel targeted cancer therapeutic platform based on the lentiviral integrase, stimulated by integrase-derived peptides, that are specifically delivered to cancerous cells via CD24 antigen-antibody targeting. INS and INR were synthesized and humanized and anti-CD24 antibodies were fused to the lentivirus envelope. The activity, permeability, stability, solubility, and toxicity of these components were analyzed. Cell death was measured by fluorescent microscopy and enzymatic assays and potency were tested in vitro and in vivo. Lentivirus particles, containing non-functional DNA led to massive cell death (40–70%). Raltegravir, an antiretroviral drug, inhibited the induction of apoptosis. In vivo, single and repeated administrations of INS/INR were well tolerated without any adverse effects. Tumor development in nude mice was significantly inhibited (by 50%) as compared to the vehicle arm. In summary, a novel and generic therapeutic platform for selective cancer cell eradication with excellent efficacy and safety are presented.

## Introduction

DNA damage is a well-established strategy for cancer therapy and is mostly achieved by cytotoxic agents or radiation. However, drug resistance and toxicity limit the use of these tools. DNA damage in the form of double-stranded breaks (DSB) can also be caused by the integration of reversed transcribed human immunodeficiency virus (HIV) DNA into the genome [1–3] of host cells, a process mediated by viral integrase (IN). This DSB ultimately leads to apoptotic cell death [4].

Rev is a 116-residue protein required for promoting nuclear export of partially-spliced viral mRNA at the late phase of the viral life cycle to allow translation [5]. The interaction of Rev with IN [6, 7] limits the process of IN-mediated integration, thus inhibiting the formation of DSBs [8]. The IN–Rev interaction can be blocked by specific IN-derived or Rev-derived peptides [9], including the two cell-permeable peptides INR2 and INS [6]. These peptides dissociate the IN–Rev complex, thus enhancing lentiviral DNA integration into the host chromosomal DNA. Importantly, the INS peptide does not only remove Rev from the inhibitory Rev-IN complex; it also directly activates IN enzymatic activity [10].

The number of IN-mediated lentiviral DNA integrations is a matter of special interest since, in HIV-infected cells, one or two integrations can occur without inducing apoptosis [11, 12]. The presence of INR and INS peptides increases the integration significantly to about 10–13 integration events in each viral-infected cell [9, 13]. This high rate of integration triggers cell death, regardless of the specific DNA sequences that are involved [2].

CD24 is a small, heavily glycosylated protein, that is considered to be a cancer biomarker, as it is expressed mostly in cancer cells and very rarely in normal cells [14–24].

Herein, we describe a method for the IN-mediated induction of cell death, targeted to CD24-expressing cancer cells. The method includes the targeted delivery of INR and INS peptides and lentivirus particles expressing a small fragment (scFv) of human anti-CD24 antibody. The lentivirus brings its own integrase, thereby labeling the cells and sensitizing them to IN-derived cell-permeable peptide activity.

## Results

### INS and INR—in vitro characteristics

#### Stability and solubility

The solubility and stability of both peptides, INS and INR, were examined in phosphate-buffered saline (PBS) as well as in rat and human plasma. Both peptides were highly soluble, with an advantage to INS and a slight priority to PBS (Supplementary Table 1). Both peptides showed stability in rat and human plasma for several hours. INS showed 60–70% stability at ~4 h incubation, reduced to 40% after 8 h and to ~25% after 24 h. In contrast, INR was 40% stable for the entire 24 h in both types of sera (Supplementary Table 2).

#### Targeted lentivirus particles combined with INS or INR promote cell death in CD24-expressing cancer cells

**Fig. 1:**
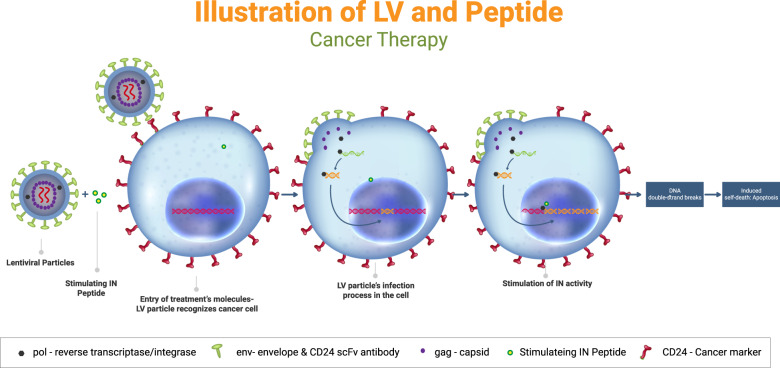
Schematic illustration of the therapeutic approach. CD24-targeted lentiviral particles selectively infect CD24-expressing tumor cells. IN-derived cell-permeable peptides enter the cells. Integrase is incorporated into the cells and is stimulated by the IN-derived peptides. The enhanced activation of the integrase leads to an increased number of DNA DSBs and ultimately to apoptotic death.

To test the effect of the lentiviral particles combined with INS or INR peptides on CD24-expressing cancer cells, several human cancer cell lines were examined. The assumed therapeutic approach is illustrated in Fig. 1. Specifically, CD24-targeting lentiviral particles are hypothesized to attach to CD24 expressing cells via antibody-antigen recognition. IN-derived cell-permeable peptides freely enter the cells, after which the integrase would be incorporated and stimulated by the IN-derived peptides. The enhanced activation of the integrase is hypothesized to lead to an increased number of DNA DSB and, ultimately, to apoptotic death.

**Fig. 2:**
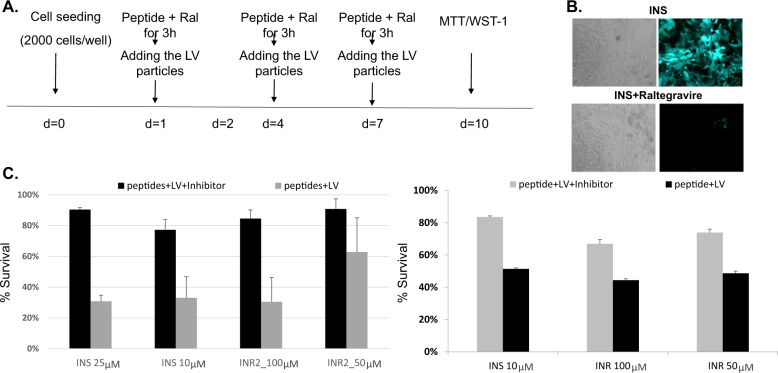
The effect of IN-derived peptides on cell survival. **A** Schematic illustration of the treatment protocol. **B** Microscopic observation of the GFP incorporation inhibition by Raltegravir. H1975 lung cancer cells were exposed to 30 MOI LV-CD24 and 25 µM INS with or without the addition of 50 µM Raltegravir. Images were taken 24 h post-treatment. **C** 2000 Panc-1 (left) and H1975 (right) cells were seeded in 96-well plates and were treated with the different items as described. Cell survival was measured by the enzymatic WST-1 (left) and MTT assays (right). % Survival was calculated as the percentage of living cells remaining after treatment with respect to non-treated cells.

Pancreatic (Fig. 2C, left), lung (Fig. 2C, right), breast and colorectal cancer cells were exposed in accordance with the treatment scheme presented in Fig. 2A. Cells were treated three times over ten days.

The combined treatment of CD24-targeted lentivirus particles together with INS or INR2 peptides led to pancreatic (Fig. 2C, left, black bars) and lung (Fig. 2C, right, black bars) cancer cell death, as demonstrated by WST-1 (Fig. 2C, left) and MTT (Fig. 2C, right) enzymatic assays. Raltegravir, an FDA-approved integrase inhibitor that acts as an anti-retroviral drug, successfully suppressed the observed cell death (Fig. 2C, gray bars).

As can be seen in Fig. 2B (upper panel), INS-treated cells showed a very high intensity of green fluorescent protein (GFP) fluorescence, which was nearly eliminated by the IN inhibitor, Raltegravir (Fig. 2B, lower panel).

#### Cytotoxicity and safety

**Fig. 3:**
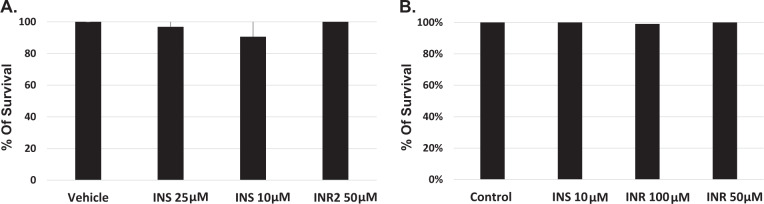
Cytotoxicity of IN-derived peptides. **A** Lymphocytes from healthy volunteers were treated with INS or INR2 three times over ten days. Cells were then analyzed for potential toxicity using the quantitative cell viability WST-1 assay. **B** H1975 lung cancer cells were treated with INS or INR2 three times over ten days and cells were then analyzed for toxicity using an MTT assay.

The potential for cytotoxicity of the IN-derived peptides in the absence of the lentiviral particles was examined in cancer cell lines and primary cells. H1975 lung cancer cells and primary lymphocytes, obtained from healthy volunteers, were treated with INS or INR three times over the course of ten days and were analyzed for potential toxicity using the quantitative cell viability MTT or WST1 assays. The results, presented in Fig. 3A, clearly show that INS and INR had no effect on lung cancer cell viability. The viability of primary lymphocytes, isolated from peripheral blood of healthy volunteers (Fig. 3B), was not affected by the presence of the IN-derived peptides either. Similar results were obtained for pancreatic (Panc-1) cells (data not shown).

### In vivo safety of the lentivirus and IN-derived peptides system

**Fig. 4:**
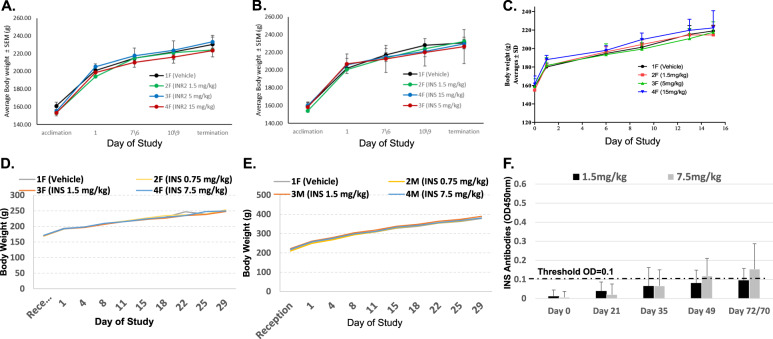
Monitoring of rat body weight during the course of MTD study. **A** Body weight was monitored after a single IV injection of INR. **B** Body weight was monitored after a single IV injection of INS. **C** Body weight was monitored after an SC injection of INS. **D** Repeated dose, IV administration of INS, at three escalating doses was performed in female rats. **E** Repeated dose, IV administration of INS, at three escalating doses was performed in male rats. **F** Rats were exposed to INS peptide by biweekly administrations over ten weeks. Blood was collected before (baseline) and after immunization (exposure) at the indicated time points. Serum was used for ELISA.

A single-dose acute toxicity study of the IN-derived peptides was performed in Sprague Dawley (SD) female rats, either by intravenous (IV) or subcutaneous (SC) administration. No clinical abnormalities were observed in any of the animals. All animals gained weight normally under both treatment administration routes, IV and SC (Fig. 4A–C, respectively). At termination, gross pathology evaluation revealed no abnormality.

IV administration of both INR (Fig. 4A) and INS (Fig. 4B) revealed that both peptides were well-tolerated up to a dose level of 15 mg/kg, the highest dose tested, with no abnormalities and normal weight gain. Similar results were obtained after SC administration of INS (Fig. 4C).

Further testing was focused on INS only, based on both the increased stability of INS (60–70% at 4 h incubation, Supplementary Table 2) as compared to INR (40%, Supplementary Table 2), as well as its dual facilitatory impact on the lentiviral integrase.

### INS—additional characteristics

#### The INS peptide directly stimulates the enzymatic activity of integrase in vitro

**Fig. 5:**
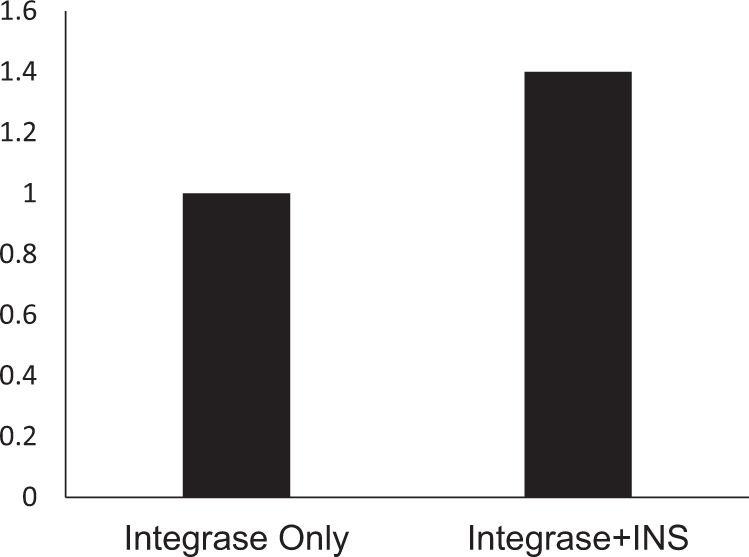
Enzymatic activity of the lentiviral integrase. Colorimetric detection of the strand-transfer recombination reaction of the lentiviral integrase before and after the exposure to the INS peptide. Sodium azide was used as a positive control compound that inhibits lentiviral integrase catalytic activity.

The direct stimulation of the enzymatic lentiviral integrase activity by the INS peptide was tested using the HIV integrase assay kit, according to the manufacturer’s instructions (ExpressBio, cat# EZ-1700, for details see Materials and Methods). The addition of the INS peptide resulted in increased activity of the integrase by 40% (Fig. 5).

**Fig. 6:**
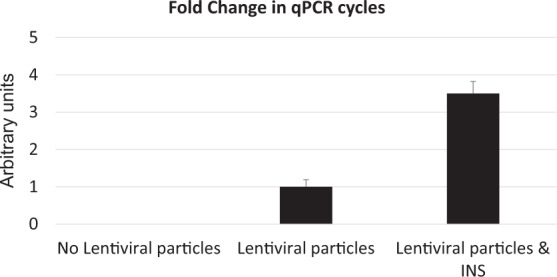
INS peptide increases the number of integration events. Fold change in qPCR cycles. H1975 cells were exposed to the indicated treatments and collected 72 h after the CD24-LV infection. Alu-Gag qPCR was performed to evaluate changes in the integration events.

The INS peptide was able to stimulate the integration of the viral DNA by targeted lentiviral particles approximately threefold, 72 h post-infection (Fig. 6). As HIV DNA integration occurs with only one or two copies of the viral genome [1], we estimate that the INS peptide increased integration events to ~4–7 integrations.

#### Toxicity and genotoxicity of repeated INS injections

In addition to the cytotoxicity assessments described above, the INS peptide was further examined in vivo in a 28-day repeated dose toxicity study with 4 weeks of repeated dosing (twice weekly) and 4 weeks of recovery, in female (Fig. 4D) and male (Fig. 4E) SD rats, via slow IV bolus. No INS-related adverse effects were seen at any of the examined dose levels, leading to a no-observed-adverse-effect level (NOAEL) of INS of 7.5 mg/kg, the highest dose tested.

The genotoxicity of the INS peptide in the absence of the lentiviral particles was examined in vitro by testing for clastogenic potential in a chromosomal aberration assay using Chinese hamster ovary (CHO-WBL) cells in the presence or absence of activation systems. No significant increases were observed in the percentage of cells with structural aberrations compared to the negative control (Table 1).

**Table 1:** Genotoxicity assessment of INS.

Treatment	±S9	Treatment time (h)	Cytotoxicity (%)^a^	Total cell scored	Cells with polyploidy (%)	Cells with endoreduplication (%)	% Gaps	% Structural aberration without gaps	% Structural aberrant cells^b^
5% acetic acid in sterile water for injection	+S9	3	0	300	0	0.3	1.7	2.3	2
Untreated Control	+S9	3	6	300	0	0.3	0.3	0.3	0.3
*INS* (µg/mL)									
10	+S9	3	5	300	0.0	0.0	0.3	1.0	1.0
25	+S9	3	−1	300	0.0	0.3	0.3	1.0	1.0
50^c^	+S9	3	46	300	0.0	7.3	0.3	1.7	1.7
CP 2.5	+S9	3	50	100	0.0	3.0	4.0	47.0	35.0*
5% acetic acid in sterile water for injection	−S9	3	0	300	0	0	0.7	0.3	0.3
Untreated Control	−S9	3	−2	300	0	0	0.3	0.7	0.7
*INS* (µg/mL)									
10	−S9	3	4	300	0.0	0.0	0.7	1.3	1.3
25	−S9	3	−10	300	0.0	0.3	0.3	1.0	1.0
50^c^	−S9	3	−7	300	0.0	0.0	0.7	1.3	1.3
MMC 0.5	−S9	3	39	100	0.0	1.0	5.0	52.0	37.0*
5% acetic acid in sterile water for injection	−S9	22	0	300	0	0.3	0.3	0.7	0.7
Untreated Control	−S9	22	−3	300	0	0	0.3	0.7	0.7
*INS* (µg/mL)									
10	−S9	22	−1	300	0.0	0.0	0.7	1.0	1.0
25	−S9	22	−3	300	0.0	0.0	0.0	1.0	1.0
50^c^	−S9	22	−1	300	0.0	0.0	0.3	1.3	1.3
MMC 0.25	−S9	22	48	100	0.0	1.0	8.0	112.0	63.0*

Treatment: CHO-WBL cells from all treatment conditions were harvested 22 h after the initiation of the treatments.

CP cyclophosphamide monohydrate, MMC mitomycin C.

%Structural Aberrant Cells: *p ≤ 0.05; using Fisher’s Exact test.

^a^Cytotoxicity was based on cell growth inhibition, relative to the concurrent negative control.

^b^Does not include cells with only gaps.

^c^Precipitate was observed at the beginning and the end of the treatment period.

There was no obvious increase in the number of cells with polyploidy or endoreduplication at any concentration in the non-activated treatment series when compared to the negative control. The INS peptide increased the number of cells with endoreduplication only at the highest dose in the S9-activated treatment series compared to the negative control. The peptide was thus concluded to be negative for the induction of structural chromosomal aberrations in CHO-WBL cells. The cytotoxicity observed in CHO-WBL cells was also low, at all tested concentrations (−S9; Table 1).

#### Immunogenicity of INS

The immunogenicity of the INS peptide, at doses of 1.5 and 7.5 mg/kg, was analyzed by evaluating the presence of anti-peptide antibodies in the serum of SD rats, using a specifically developed enzyme-linked immunoassay (ELISA) assay. Rats were exposed to INS peptide by biweekly administrations over the course of 10 weeks. No morbidity or mortality related to the INS peptide was observed at the tested doses during the in-life period. All animals gained weight normally with no statistical differences between the study groups. The ELISA results showed no or very low immunogenicity at the dose levels tested of 1.5 and 7.5 mg/ml, respectively (Fig. 4F).

#### CD24-targeted lentiviral particles effectively inhibit tumor growth in vivo in combination with the INS peptide

**Fig. 7:**
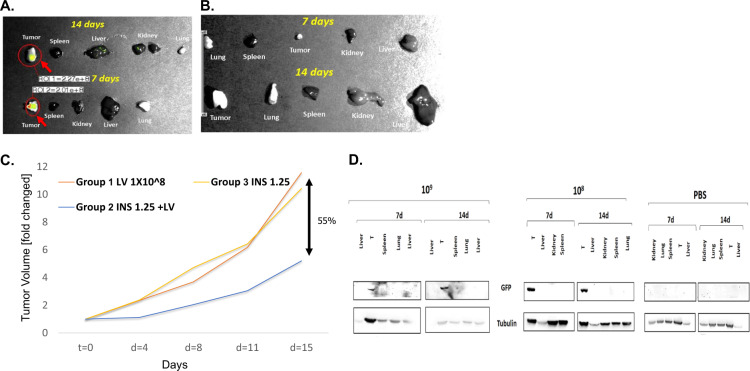
In vivo evaluation of INS. Live imaging (IVIS device) of tumors and selected organs were performed 7 and 14 days after systemic injection. **A** Organs following injection of lentiviral particles. **B** Organs and tumor following injection of PBS. **C** INS inhibited lung tumor development. Human 1975 lung cancer cells (5 × 10) were injected subcutaneously, at one site on the back of athymic nude mice. Mice were treated IP, with CD24-lentivirus particles (1 × 10, orange), INS (1.25 mg/kg, yellow), or the combination of them (blue). The graph shows representative results. **D** Western blotting confirmed the presence of GFP only in tumor tissue (indicated with T), and not in other tissues, at both doses (10 and 10 particles). No GFP was detected in the control group (PBS). Tubulin was used as a loading control.

To confirm the ability of the targeted lentiviruses to reach and infect the tumor cells after systemic administration, intraperitoneal injections were performed on nude mice bearing xenografts derived from CD24-positive H1975 lung cancer cells. One and two weeks after injecting the lentiviruses, the expression of the GFP was estimated by imaging (Fig. 7A, B) and western blot analysis (Fig. 7D).

Using both methods 7 and 14 days after injection, the GFP was highly expressed in the tumor, while hardly detectable in the other tested tissues of the treated mice (Fig. 7A, B), indicating that the lentivirus-INS system delivery was successfully confined to the target tissue. Fourteen days after injection, some staining in the kidney and liver was evident, probably due to excretion processes.

Following the demonstration of targeted delivery, the efficacy of the suggested system was evaluated in the same mouse model of lung cancer (Fig. 7C). Effective inhibition of tumor growth was achieved using the INS peptide (1.25 mg/kg) that was injected, along with the CD24-targeted lentiviral particles, twice weekly for two weeks. 55% reduction in tumor volume was observed in the treated group compared to mice that received the lentiviral particles or the peptide alone. In addition, no toxicity or any adverse effects were observed throughout the entire study.

## Discussion

In this study, we provide proof of concept for a new strategy for the effective and selective elimination of human pancreatic and lung cancer cells in vitro and in animal models.

This novel platform is comprised of the following unique elements: (1) lentiviral particles that carry non-functional DNA (only a GFP cassette for infection validation); (2) an scFv against CD24 fused to the lentivirus envelope (VSV-G) for targeted delivery to CD24-expressing cancer cells; (3) IN-derived peptides (INS or INR) that stimulate non-functional DNA integration into the cell genome, which in turn increases the number of DSBs, thus stimulating cancer cell death. Indeed, the use of this system induced massive lung and pancreatic cancer cell death in vitro. In vivo targeting of SC lung-derived tumors, by LV-scFvCD24 together with the IN-derived peptides, lead to significant (>60%) inhibition of tumor growth. Normal cells, which typically do not express CD24, remained uninfected.

It is important to stress that, unlike other gene therapy/delivery approaches, the suggested strategy does not contain any functional DNA element. No viral DNA is inserted into the infected cell’s genome. In this system, the virus acts as both a carrier and an oncolytic, which vastly differs from other forms of gene therapy. Although the non-functional DNA integration may disrupt gene expression, most likely that this cancer cell will undergo apoptosis and die, due to multiple integration events leading to genome instability. Currently, we are developing the next generation platform, consisting of a new lentiviral vector that will comprise both parts, i.e., it would carry the INS peptide and be targeted only to cancer cells by means of an anti-CD24 scFv.

This is a proof of concept for a new universal cancer therapy approach, as the lentiviral compound can be engineered to express almost any scFv (target moiety) according to the antigens that are selectively expressed on the targeted cancer cells and not on normal cells.

Our results demonstrate an excellent safety profile of the proposed system. Several factors substantially reduce the likelihood of developing toxicity in humans, including (a) IN-derived peptides affect only viral proteins and not human ones. Indeed, ex vivo, these peptides were not toxic to lymphocytes derived from healthy volunteers; (b) the target (CD24) is expressed almost exclusively in cancer cells; (c) the lentiviral vectors are self-inactivating and were shown to exert no genotoxicity in 43 gene therapy studies [25]. Moreover, our targeted LV particles lack a strong HIV-1 LTR promoter, resulting in a reduced tendency to activate nearby proto-oncogenes [26]. The genetic safety of the IN-derived peptides, both in acute and repeated injections to rats, was negligible. No significant increases were observed in the percentage of cells with structural aberrations as compared to cells treated with either cyclophosphamide monohydrate or mitomycin C. INS and INR peptides were found to be non-toxic as the MTD and repeated dose studies demonstrated that a single IV or SC administration of the peptides in female rats was well tolerated and did not cause any significant adverse clinical effects compared to the vehicle treatment (acetate isotonic buffer).

Numerous different peptides derived from the HIV-1 integrase were tested. INR and INS were found to have the strongest potency to kill HIV-infected cells [10]. We chose to further continue with the INS peptide not only because of its ability to dissociate the REV-IN complex but also due to its potency to further stimulate IN activity by binding directly to it. Indeed, the impact of the lentivirus-INS system via the induction of genomic instability and programmed cell death was confirmed by blocking this cascade through the IN inhibitor Raltegravir.

INS is expected to be effective, as its solubility and stability were confirmed in PBS, in rats, and also in the plasma of humans, and was found to be somewhat advantageous as compared to INR. In addition, we found that the viability of primary lymphocytes, isolated from peripheral blood of healthy volunteers, as well as the viability of the human cancer cells, were not affected by the presence of the INS peptide. These results are supported by previously published work done on HeLa cells showing that INS is cell-permeable and non-toxic [10].

Furthermore, very low immunogenicity was observed in the tested dosages, which are higher than the clinical dosage.

The compounds (CD24-targeted LV and INS peptide) were given to four terminally ill patients with metastatic end-stage cancer, who failed all therapies as compassionate use. The patients were followed carefully. No clinical or laboratory toxicity was seen. It is difficult to estimate efficacy in this setting, but in the first patient, who received the drug for ~3 weeks, the clinical picture of intestinal obstruction improved and one of the liver metastases was resolved.

The suggested novel therapeutic strategy is safe, should be effective, and with appropriate modifications, can be used in almost any cancer type.

## Material and methods

### Cell lines

H1975 human lung adenocarcinoma, Panc-1 human pancreatic cancer, and HT29 human colon cancer cell lines were grown in high-glucose Dulbecco’s modified Eagle’s medium (DMEM), all supplemented with 5% heat-inactivated fetal bovine serum (FBS), 1% penicillin and streptomycin in an atmosphere of 95% oxygen and 5% CO_2_. The different cell lines were obtained from the ATCC.

### Peptide synthesis and purification

Peptides, INS, and INR2 (Supplementary Table 3) were synthesized by Pepscan Presto (Lelystad, Netherlands) or by Wuxi AppTec (Wuhan) Co., or by Polypeptide (San Diego, US).

### Determination of IN activity

The IN enzymatic activity was evaluated using the HIV integrase assay kit according to the manufacturer’s instructions (ExpressBio, cat# EZ-1700). Briefly, Streptavidin-coated 96-well plates were coated with a double-stranded HIV-1 LTR U5 donor substrate (DS) DNA containing end-labeled biotin. Full-length recombinant HIV-1 IN protein was loaded onto the DS DNA substrate. The tested items were added to the enzyme reaction and then a different ds target substrate (TS) DNA containing a 3′-end modification was added to the reaction mixture. The HIV-1 integrase cleaves the terminal two bases from the exposed 3′-end of the HIV-1 LTR DS DNA and then catalyzes a strand-transfer recombination reaction to integrate the DS DNA into the TS DNA. The products of the reaction were detected calorimetrically using an HRP-labeled antibody directed against the TS 3′-end modification. Sodium azide was used as a positive control compound that inhibits HIV-1 integrase catalytic activity.

### Solubility and stability test of IN derived peptides

#### General

Analytic HPLC-MS was performed using an Agilent 1260 series Liquid Chromatography/Mass Selective Detector (MSD) equipped with an electrospray interface and a UV diode array detector. Analyses were performed on an ACE C8, 50 × 3.0 mm, 3 µm column, with an isocratic 0.1% formic acid/ACN eluent, at a rate of 1 ml/min over a period of 5 min. UV absorption was measured at 280 nm, 220 nm, and 215–395 nm. MS analysis was performed with ESI (electron spray ionization) with a positive ionization method.

#### Plasma stability and solubility

Four reaction systems were prepared; INS and INR2 in human plasma, INS and INR2 in rat plasma (Supplementary Table 2). Freshly prepared 10 mg/ml stock solution of the peptides was injected into plasma or PBS solution (1:1). The mixture was diluted with methanol to precipitate the plasma proteins. The samples were filtered through PTFE 0.45 µm filters and run in LC-MS. Samples were taken at the following times: 0, 15, 30, 60, 120, 240, and 480 min and after 24 h.

Plasma solubility was determined at 0 min and was verified at 60 min (if no degradation was observed).

### Estimation of cell viability using the MTT or WST-1 assay

#### Cancer cells

The cell-killing effect of the system was measured by the MTT3-(4,5-dimethylthiazol-2-yl)-2,5-diphenyltetrazoliumenzymatic assay or by 2-(4-iodophenyl)-3-(4-nitrophenyl)-5-(2,4-disulfophenyl)-2H-tetrazolium (WST-1) assay. Two thousand cultured cells were seeded onto 96-well plates (in culture media, DMEM, or RPMI). After 24 h, different concentrations of the diverse peptides (and inhibitors) were added for 3 h. Thirty MOI of the targeted lentiviral particles were added to the cells and the plates centrifuged for 90 min at 800×*g*, 20 °C. Three and six days later, this procedure (peptides/inhibitor and LV treatments) was repeated. On the tenth day, cell viability was analyzed either by MTT or by WST-1; the media was replaced by fresh media (100 μl per well) containing 1 mg/ml MTT, and the cells were incubated for 2–4 more hours. MTT-formazan crystals were dissolved by the addition of extraction solution (0.1 N HCl in absolute isopropanol). Absorbance at 570 nm and a reference wavelength of 690 nm was recorded on an automated microplate reader. For WST-1, media was replaced and WST-1 was added directly to the culture wells followed by incubation of 10–60 min at 37 °C. Plates were read by an automated microplate reader by measuring the absorbance of the dye with a wavelength of 450 nm and a reference wavelength of 630 nm.

### Alu-Gag HIV PCR

Quantification of the integrated DNA of lentiviral particles was examined by repetitive-sampling Alu-HIV PCR, according to the protocol “quantification of integrated HIV DNA by Alu-HIV PCR” [25–27]. The sequences of both PCR primers (Supplementary Table 4) were examined and found compatible with the used lentiviral particles. Six replicates were used for each sample.

### Cytotoxicity

Written informed consent was obtained from all eligible participants prior to entry into the study. Approval for this study was provided by Kaplan Medical Center’s Ethics Committee (Protocol No. ZMS1/2016). Lymphocytes of healthy volunteers were isolated from the peripheral blood by Ficoll-Paque density gradient centrifugation. Cells were incubated in DMEM with different peptide concentrations for 72 h. Then, 100 µl of cells were taken for toxicity testing using a quantitative cell viability test, the WST-1 assay.

H1975 lung cancer cells were treated three times over the course of 10 days (on days 2, 4, and 7) with different concentrations of the tested peptides. Following ten days of incubation, cells were analyzed for potential toxicity using a quantitative cell viability test, the MTT assay.

### Genotoxicity

Genotoxicity was examined by testing for clastogenic potential in a chromosomal aberration assay using Chinese hamster ovary (CHO-WBL) cells in the absence or presence of activation systems for 3 and 22 h, and in the presence of an activation system (Aroclor 1254 induced rat liver S9) for 3 h. All cells were cultured for 22 h after initiation of INS treatment.

### Safety evaluation following a single dose administration of (1.5, 5, and 15 mg/kg) of INR2 and INS to SD rats

Animals were fed a commercial rodent diet (Teklad Certified Global 18% Protein Diet) ad libitum. Animals had free access to sterilized and acidified drinking water (pH between 2.5 and 3.5). SD rats were randomly allocated to cages. The maximum tolerated dose (MTD) of a single dose of the INS peptide was evaluated by IV injection or by SC injection at a dose volume of 5 ml/kg (1.5–15 mg/kg), in three rats for each group. Also, the MTD of a single dose of the INR2 peptide was evaluated by IV injection for the same dosages.

The following study variables and endpoints were examined:

- Mortality and morbidity—morbidity and mortality check was performed at least twice daily.
- Bodyweight measurements—during acclimation, before dosing, and weekly thereafter.
- Detailed clinical observation—prior to dosing, frequently for the first four hours post-dosing and once a week thereafter.
- Necropsy and gross pathology—macroscopic findings on all study animals with special attention to changes in thymus, spleen, and lymph nodes.
- Organ weight post-mortem and tissue preservation—at the termination day, selected tissues were weighed and embedded in formalin.

#### Twenty-eight days repeated dose toxicity study

Four weeks of repeated dosing followed by four weeks of recovery was performed in female and male SD rats, via slow IV bolus of the INS peptide. Eighty rats (40 males and 40 females) were utilized for the main study groups (*n* = 10 per study group). An additional 40 animals (20 males and 20 females) were used as recovery groups (*n* = 5 per study group). Group allocation was done without investigator blinding. Study evaluations included: mortality and morbidity, body weight, food consumption, clinical signs, ophthalmological observation, blood collection for hematology, clinical chemistry, coagulation, urine analysis, necropsy, and gross pathology, organ weight, and histopathology. The study was approved by the Institutional Review Board of Tel Aviv Sourasky Medical Center.

### Lentivirus production

High-scale production of the targeted lentiviruses (CD24 scFv antibody derivatives-fused lentiviral envelope) was performed by Sirion Biotech GmbH (Martinsried, Germany). Non-functional DNA or GFP expressing lentiviral particles (maps in Fig. S1) with packaging containing a single-chain antibody directed against CD24 as N-terminal fusion to VSVG (map in Fig. S1) were produced. The uptake of the targeted LV particles by different target cells was examined by several methods such as microscopic observation, FACS analysis, etc.

### In vivo efficacy evaluation of the combination of IN derived peptide together with CD24-targeted LVs

In order to assess the combination of the above peptides with the targeted viruses on tumor development, human lung cancer cells (5 × 10^6^) were injected subcutaneously at one site on the back of male 6–8 weeks old athymic nude mice. When tumors were palpable (~0.3–0.5 cm^3^), the mice were randomly divided into groups and the different treatments (Supplementary Table 5) were injected IP, twice a week for 15 days. According to common practice, should no tumor develop in an animal, then it would have been excluded from the experiment. The experiment was repeated three times. Group allocation was done without investigator blinding. The study was approved by the Institutional Review Board of Tel Aviv Sourasky Medical Center.

### Immunogenicity

Animals were fed ad libitum a commercial rodent diet (Teklad Certified Global 18% Protein Diet). Animals had free access to sterilized and acidified drinking water (pH between 2.5 and 3.5). SD rats were randomly allocated to cages and treated twice a week, for ten weeks with INS peptide, at two doses of 1.5 and 7.5 mg/kg. Rat blood was collected before immunization (exposure to the peptide) to serve as a baseline, and after immunization at day 21, 35, 49, and 70/72. The immunogenicity was analyzed by evaluation of anti-peptide antibodies in the sera of SD rats, using a specific developed ELISA test. A 96 well plate was coated overnight with INS labeled with biotin (5 µg/ml) (Thermo Scientific) followed by blocking (3% Milk in PBS) for 1 h at room temperature (RT). Serum samples were diluted at 1:50 and added to the plates for 1 h at RT. After washing with PBST (three times), a donkey anti-rat antibody (Jackson Laboratories, cat. no. 705-035-003) (diluted 1:5000) was added and incubated for 1 h at RT. Then, TMB, diluted 1:2 was added and the reaction was stopped after 10 min with HCL solution. Absorbance was measured by an automated microplate reader (iMARK microplate reader, BioRad) with a wavelength of 450 nm.

## Supplementary information

Supplementary Information without tables

Supplementary Table 1

Supplementary Table 2

Supplementary Table 3

Supplementary Table 4

Supplementary Table 5
