# Supplementary material for: Integrase-derived peptides together with CD24-targeted lentiviral particles inhibit the growth of CD24 expressing cancer cells
Source: Oncogene. 2021 May 6;40(22):3815–25. doi: 10.1038/s41388-021-01779-5 (PMC8175240; doi:10.1038/s41388-021-01779-5)
Supplement: Supplementary file 1 — Supplementary Information without tables [file 41388_2021_1779_MOESM1_ESM.docx]

**Supplementary Information**

Figure S1: Vector Maps (GFP, non-functional DNA/ Empty and Packaging plasmids)


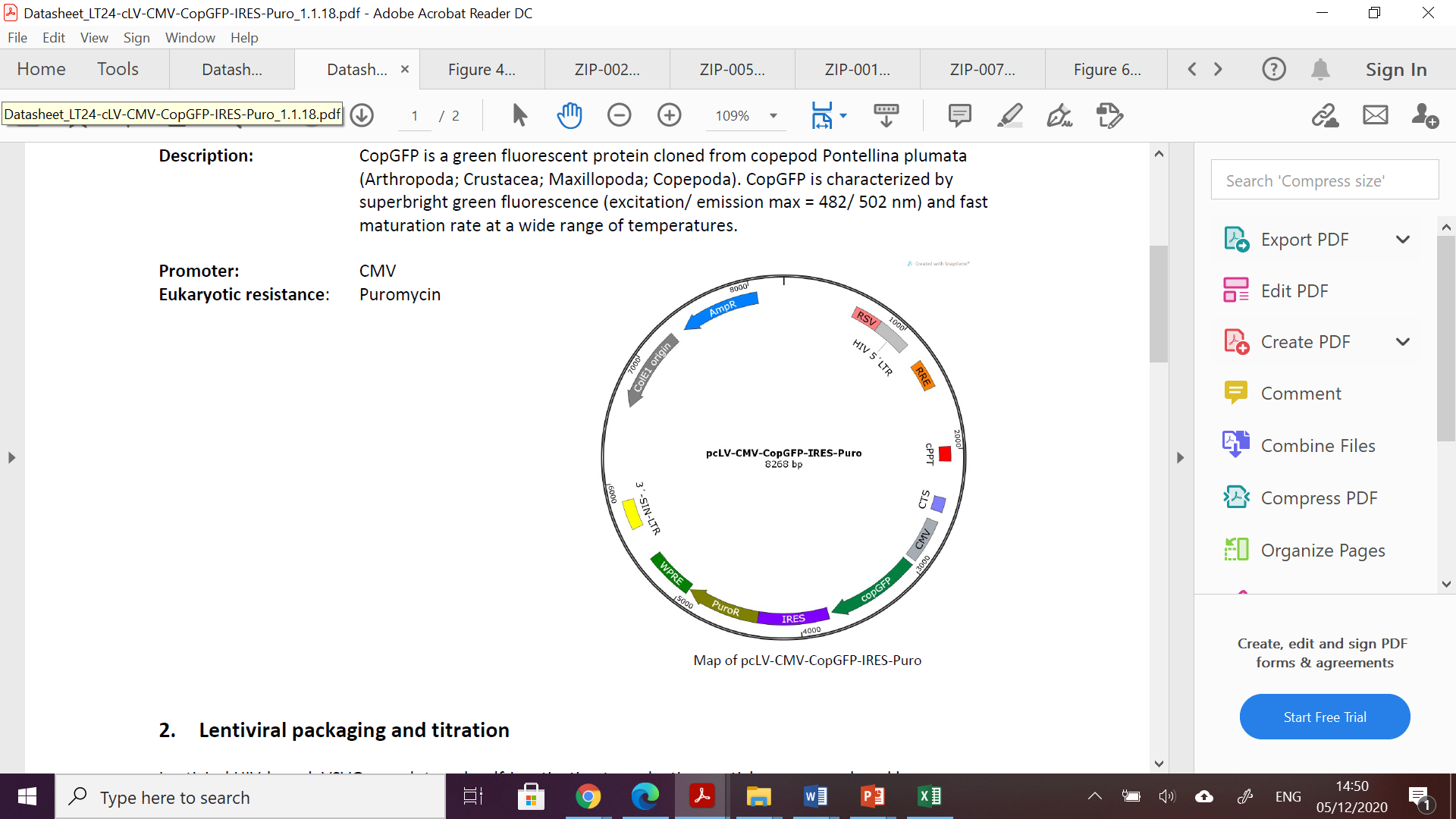

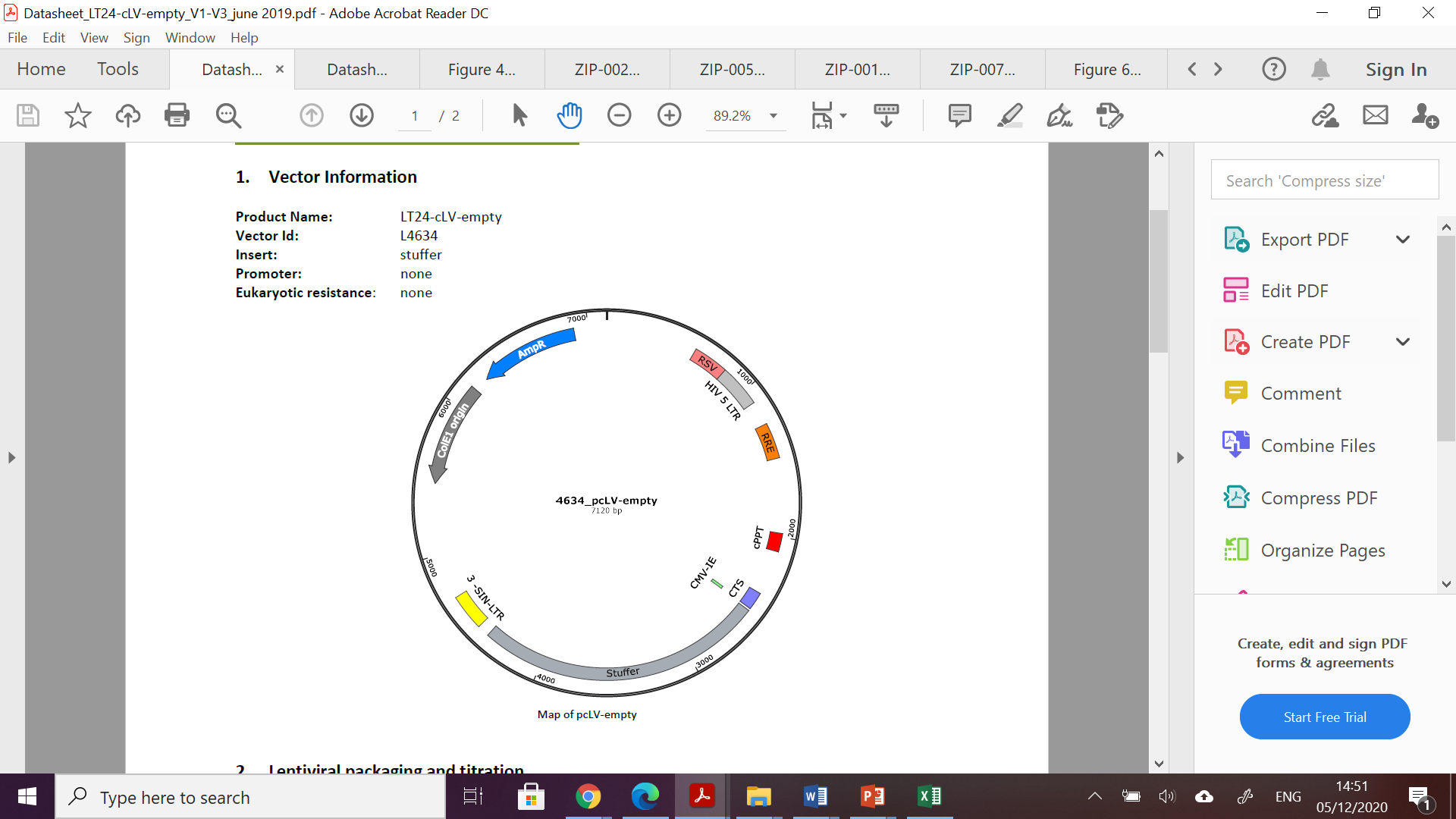


Map of pcLV-CMV-CopGFP-RES-Puro

Map of pcLV-empty


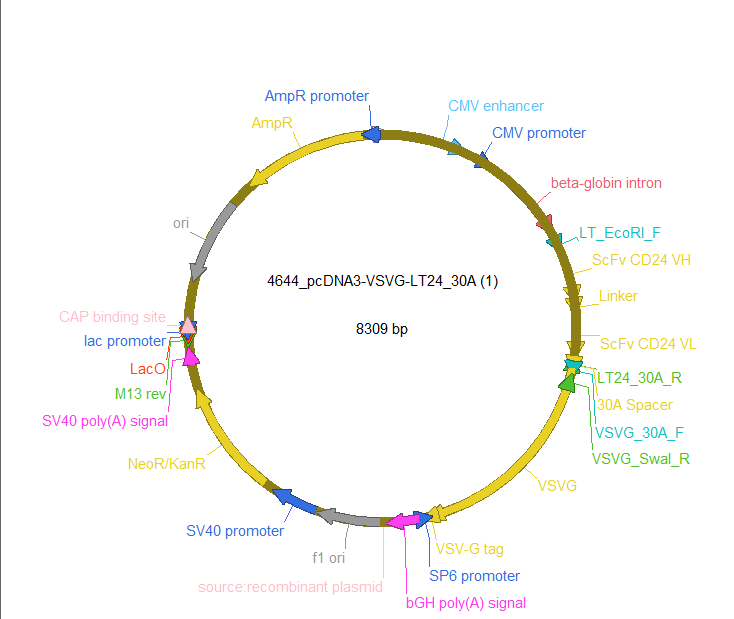


Map of pcDNA3-VSVG-LT24_30A (1)
