# Supplementary material for: Integrase-derived peptides together with CD24-targeted lentiviral particles inhibit the growth of CD24 expressing cancer cells
Source: Oncogene. 2021 May 6;40(22):3815–25. doi: 10.1038/s41388-021-01779-5 (PMC8175240; doi:10.1038/s41388-021-01779-5)
Supplement: Supplementary file 2 — Supplementary Table 1 [file 41388_2021_1779_MOESM2_ESM.docx]

Supplementary Table 1: Solubility of IN-derived peptides (from 5 mg/mL sample)

|  |  | **INS** | | **INR2** | |
| --- | --- | --- | --- | --- | --- |
|  |  | **mg/mL** | **%** | **mg/mL** | **%** |
| **PBS** |  | 4.46 | 89.2 | 3.31 | 66.2 |
| **Plasma** | Rat | 4.01 | 80.2 | 2.13 | 42.6 |
|  | Human | 3.1 | 62 | 2.21 | 44.2 |
