# Supplementary material for: Integrase-derived peptides together with CD24-targeted lentiviral particles inhibit the growth of CD24 expressing cancer cells
Source: Oncogene. 2021 May 6;40(22):3815–25. doi: 10.1038/s41388-021-01779-5 (PMC8175240; doi:10.1038/s41388-021-01779-5)
Supplement: Supplementary file 3 — Supplementary Table 2 [file 41388_2021_1779_MOESM3_ESM.docx]

Supplementary Table 2: Stability test of IN-derived peptides

| **Time [hours]** | **Area [mAU*S]** | **Concentration calculated from LC-MS data [mg/ml]** | **Analyte Remaining %** |
| --- | --- | --- | --- |
| **INS stability in human plasma** | | | |
| 0 | 10937.1 | 3.29 | 65.7 |
| 0.25 | 10116.1 | 3.04 | 60.9 |
| 0.5 | 11952.7 | 3.59 | 71.1 |
| 1 | 9701.31 | 2.92 | 58.4 |
| 2 | 10420.4 | 3.13 | 62.7 |
| 4 | 10344.9 | 3.11 | 62.2 |
| 8 | 7293.32 | 2.21 | 44.2 |
| 24 | 3836.77 | 1.19 | 23.8 |
| **INR stability in human plasma** | | | |
| 0 | 8797.68 | 2.05 | 40.9 |
| 0.25 | 8671.25 | 2.02 | 40.3 |
| 0.5 | 9733.44 | 2.27 | 45.3 |
| 1 | 10161.1 | 2.37 | 47.3 |
| 2 | 8428.21 | 1.96 | 39.2 |
| 4 | 8859.97 | 2.06 | 41.2 |
| 8 | 8237.25 | 1.91 | 38.3 |
| 24 | 8374.91 | 1.95 | 38.9 |
| **INS stability in Rat plasma** | | | |
| 0 | 13401.1 | 4.01 | 80.3 |
| 0.25 | 11358.5 | 3.41 | 68.2 |
| 0.5 | 10862.1 | 3.26 | 65.3 |
| 1 | 9770.38 | 2.94 | 58.9 |
| 2 | 8361.02 | 2.53 | 50.5 |
| 4 | 6335.57 | 1.93 | 38.6 |
| 8 | 5908.86 | 1.8 | 36.1 |
| 24 | 2311.42 | 0.74 | 14.8 |
| **INR stability in Rat plasma** | | | |
| 0 | 8979.24 | 2.09 | 41.8 |
| 0.25 | 10978.3 | 2.56 | 51.1 |
| 0.5 | 7382.13 | 1.71 | 34.3 |
| 1 | 9341.65 | 2.17 | 43.5 |
| 2 | 9380.04 | 2.18 | 43.6 |
| 4 | 8224.94 | 1.91 | 38.2 |
| 8 | 7590.82 | 1.76 | 35.3 |
| 24 | 7933.39 | 1.84 | 36.9 |
