# Supplementary material for: Integrase-derived peptides together with CD24-targeted lentiviral particles inhibit the growth of CD24 expressing cancer cells
Source: Oncogene. 2021 May 6;40(22):3815–25. doi: 10.1038/s41388-021-01779-5 (PMC8175240; doi:10.1038/s41388-021-01779-5)
Supplement: Supplementary file 4 — Supplementary Table 3 [file 41388_2021_1779_MOESM4_ESM.docx]

Supplementary Table 3: IN-derived peptide sequences

| **Peptide** | **Sequence** |
| --- | --- |
| INS | WTAVQMAVFIHNFKRK |
| INR | WGSNFTSTTVKA |
