# Supplementary material for: Integrase-derived peptides together with CD24-targeted lentiviral particles inhibit the growth of CD24 expressing cancer cells
Source: Oncogene. 2021 May 6;40(22):3815–25. doi: 10.1038/s41388-021-01779-5 (PMC8175240; doi:10.1038/s41388-021-01779-5)
Supplement: Supplementary file 5 — Supplementary Table 4 [file 41388_2021_1779_MOESM5_ESM.docx]

Supplementary Table 4: Alu-Gag PCR’s primers

| **Primers** | **Sequence** |
| --- | --- |
| 1 st PCR (Alu-Gag PCR) | |
| Forward primer Alu | 5’-GCCTCCCAAAGTGCTGGGATTACA-3’ |
| Reverse primer Gag | 5’-GTTCCTGCTATGTCACTTCC-3’ |
| 2 nd PCR (qPCR) | |
| Forward primer RU5 (R Forward) | 5’-TTAAGCCTCAATAAAGCTTGCC-3’ |
| Reverse primer RU5 (U5 Reverse) | 5’-GTTCGGGCGCCACTGCTAGA-3’ |
| Probe | 5'-CCAGAGTCACACAACAGACGGGCACA-3' |
