# Supplementary material for: Integrase-derived peptides together with CD24-targeted lentiviral particles inhibit the growth of CD24 expressing cancer cells
Source: Oncogene. 2021 May 6;40(22):3815–25. doi: 10.1038/s41388-021-01779-5 (PMC8175240; doi:10.1038/s41388-021-01779-5)
Supplement: Supplementary file 6 — Supplementary Table 5 [file 41388_2021_1779_MOESM6_ESM.docx]

Supplementary Table 5: *In vivo* study design

| **Treatment** | **Group** |
| --- | --- |
| PBS | 1, n=6 |
| 1x10^8^ CD24-LV particles | 2, n=6 |
| INS 1.25 mg/kg | 3, n=6 |
| 1x10^8^ CD24-LV particles + INS 1.25 mg/kg | 4, n=6 |
